# Rescuing the Inhibitory Effect of the Salivary Gland Hypertrophy Virus of *Musca domestica* on Mating Behavior

**DOI:** 10.3390/insects14050416

**Published:** 2023-04-27

**Authors:** Marissa Gallagher, Arianna Ramirez, Christopher J. Geden, John G. Stoffolano

**Affiliations:** 1 Neuroscience Department, University of Massachusetts, Amherst, MA 01003, USA; 2 Biology Department, University of Massachusetts, Amherst, MA 01003, USA; 3 Center for Medical, Agricultural and Veterinary Entomology, USDA, Agricultural Research Service, Gainesville, FL 32608, USA; 4 Stockbridge School of Agriculture, University of Massachusetts, Amherst, MA 01003, USA; stoff@umass.edu

**Keywords:** juvenile hormone, octopamine, methoprene, corpus allatum, sesquiterpenoids, hormone supplemental rescue therapy, mating receptivity

## Abstract

**Simple Summary:**

House flies have been global pests of humans and animals since antiquity, and are notoriously difficult to control. Flies in nature are sometimes infected with salivary gland hypertrophy virus (*Md*SGHV), which prevents them from mating or laying eggs. A better understanding of how the virus works could be helpful for through use as a fly management tool. In this study, we found that infected female flies, which normally do not mate, could be induced to mate by treating them with hormones that are involved in normal fly reproduction. The results provide insight into the mechanisms by which the virus tricks the fly into being unresponsive to male suitors.

**Abstract:**

Infection with salivary gland hypertrophy virus (*Md*SGHV) of *Musca domestica* prevents female flies from accepting copulation attempts by healthy or virus-infected males. This study focused on supplemental hormonal rescue therapy for mating behavior in virus-infected female house flies. The inhibitory effect of the virus on mating behavior in females injected with *Md*SGHV was reversed by hormonal therapy in the form of octopamine injections, topical application of methoprene, or both therapies combined along with 20-hydroxyecdysone. Infected females whose mating responsiveness had been restored continued to have other viral pathologies associated with infection such as hypertrophy of the salivary glands and a lack of ovarian development.

## 1. Introduction

Mating behavior is essential for those insects that rely on the successful transfer of both viable sperm and female egg development. Without either, individuals have wasted gametes. Various factors have been shown to influence normal mating in insects. One factor that is currently under investigation is the effect of viruses on either sperm/egg production or on mating behavior. Studies on the nematode, *Caenorhabditis elegans*, demonstrated that virus infection somehow changes male mating choice [1]. One of the most complete studies showing the effect of a virus on insect mating behavior is that of Burand et al. [2], who showed that the virus Hz-2v altered mating behavior and pheromone production in female moths. The review by Kariithi et al. [3], in addition to focusing on tsetse flies, provides information that diverse viruses of insects, including dipterans, affect both male and female reproductive systems. 

Hytrosaviruses are a relatively recently discovered group of viruses that are mostly known from forms that infect house flies and *Glossina* species [4]. They are double-stranded DNA, enveloped viruses that are characterized by causing hypertrophy of the salivary glands and effects on the reproductive system. The virus infecting house flies (*Md*SGHV) is thought to be transmitted per os when infected flies deposit the virus on food and has been regarded as a potential biological control agent [5,6]. In contrast, the viruses infecting *Glossina* spp. are mainly viewed as an impediment to tsetse mass-rearing efforts for releases in sterile insect technique programs [7].

Coler et al. [8] first reported that *Md*SGHV shuts down ovarian development in house flies. They did not, however, mention the effect of the virus on mating behavior for either sex. Later, Leitze et al. [9] reported on mating trials using different combinations of healthy versus infected males and females at different times post-infection. They demonstrated that females virally infected for 72 h, post-eclosion at the previtellogenic stage, had almost zero percentage of copulation when paired with healthy males. They suggested that the virus somehow influenced the central nervous system, thus shutting down mating receptivity.

To explain the effect of the virus on mating receptivity, Kariithi et al. [10] provided evidence that low hemolymph sesquiterpenoid levels may account for the female’s refusal to mate. They reported that “*Md*SGHV replication in the CA/CC [corpus allatum/corpus cardiacum] complex potentially explains the significant reduction of hemolymph sesquiterpenoid levels, the refusal to mate, and the complete shutdown of ovarian development in viremic females.” They did not, however, examine the effects of biogenic amines or (S)-methoprene, a juvenile hormone (JH) mimic, both of which have previously been used by researchers to study mating behavior in flies [11,12]. In their review paper, Kariithi et al. [4] reported that hytrosavirus replicates within the CA and suggested that it disrupts JH hormone biosynthesis. 

Because our laboratory has previously studied mating behavior in flies [13,14], we decided to see if we could reverse the effect that salivary gland hypertrophy virus (*Md*SGHV) has on mating responsiveness in house flies. Compared to previous studies, a different approach for rescuing mating behavior in infected females was used here. We treated infected female house flies with two chemicals—octopamine (OA) and JH [i.e., (S)-methoprene]. OA, a biogenic amine, is a neurohormone in insects known for its involvement in fly mating [15]. (S)-methoprene, a synthetic analog of juvenile hormone (JH), has been previously shown to influence the mating behavior of flies [16]. The effects of OA and (S)-methoprene were examined separately on infected females. Our hypothesis was that mating responsiveness could be rescued in virus-infected females if they were given hormone therapy that could counteract the effects of infection.

## 2. Materials and Methods

### 2.1. Maintaining Flies

Flies were from the WTF strain maintained at USDA-ARS-CMAVE in Gainesville, FL. Adults were separated upon emergence and put into separate cages based on sex. Cages (20 × 20 × 20 cm) were provided with two 30 mL plastic containers of water with saturated Absorbal^©^ wicks and one 30 mL plastic container with a 1:1 mixture of dry granulated sugar and powdered milk. Cages were held at 24–25 °C in incubators. 

The WTF house fly colony includes a small but variable proportion of females that are autogenous (do not require protein for mating receptiveness or ovarian development) in each generation. To eliminate autogenous flies from the assays, females were pre-screened for signs of autogeny by placing them for 1 h with active males (1:1 females:males) ready to mate and removing any females or males that mated from the study. Only non-autogenous females were used for the mating studies. After removing all flies suspected of being autogenous, the remaining flies were separated again into groups of males and females.

### 2.2. Infection with Virus

Female flies were infected within 24 h of emergence with the FL strain of *Md*SGHV as described by Lietze et al. [9] and Shaler et al. [17]. Briefly, frozen virus samples containing a single pair of homogenized/filtered ovaries from infected flies in 50 µL of sterile saline were thawed then serially diluted fourfold (10^−4^ dilution) in PBS. Flies were cold-immobilized and injected in the thorax with 2.5 µL of the diluted virus, resulting in injection of about 8000 viral copies based on Lietze et al. [9]. The 10^−4^ dilution was selected for infection because we had previously determined this to be the best dilution out of a series of 12-fold dilutions to consistently produce 100% infected flies with hypertrophied salivary glands and no ovarian development (unpublished data).

### 2.3. Hormone Treatments

Octopamaine (OA) treatments were administered to the females via the same injection used to deliver the virus to avoid mortality from multiple injections. Because it is soluble in PBS, OA (6 mg) was directly dissolved into the *Md*SGHV inoculum (200 μL), producing a final diluted concentration of OA (30 μg/μL). When the 10^−4^ diluted virus inoculum was injected (2.5 μL) into each cold-immobilized female, final dosages of 75 μg of OA [15] were administered per fly. 

Methoprene was applied topically. A stock solution of (S)-methoprene (5 µg/μL) was prepared by mixing methoprene (5.40 μL), density of 926.1 µg/µL, with acetone (994.60 μL). Cold-immobilized flies were treated by applying 1 μL of this solution (5 µg (S)-methoprene) to the thoracic surface of each female at 48 and 72 h after infection, resulting in a final dosage of 10 μg (S)-methoprene per fly. 

A final experimental condition was a combination of: (1) topical application of methoprene as before; (2) injection with octopamine as before; and (3) inclusion of 2.5 μg of 20-hydroxyecdysone (20E) in the initial injection along with the virus and OA.

Several sets of control flies were set up as well: (1) uninfected, untreated flies; (2) uninfected flies injected with 2.5 μL PBS; and (3) uninfected flies treated topically with 1 μL acetone. Finally, uninfected control females were also set up that were denied protein (sugar-fed only) and either left untreated or treated topically with methoprene as described previously.

For each bioassay, cages of 50 healthy male flies and 15 flies from each of the treatment or control groups were set up and provided with food and water. An additional sample of five females injected with the virus were set aside from each batch of virus-injected flies to provide a virus quality control check before mating bioassays. These flies were dissected 72 h after viral injection and examined to confirm both hypertrophy of the salivary glands and lack of ovarian development. Mating bioassays were only conducted if all of the injected flies in a batch were symptomatic for infection.

### 2.4. Mating Timeline in House Flies

To determine an appropriate timeline of mating behavior for our assays, preliminary tests were first conducted to determine when females were optimally receptive to mating attempts. To do this, 24 h-old healthy females were placed into 7 separate, 16 oz plastic containers, with water, granulated sugar, and powdered milk, and 24 h-old males were added to each cup for 7 consecutive days. Each day, when males were added, they were observed for mating behavior for 1 h. Males showed clear mating behavior attempts beginning when females were 48 h old, but females did not accept male attempts until after 120 h post-eclosion. Based on these observations, mating behavior observations were done with females that were 120 h old at the time of bioassays.

### 2.5. Observation of Mating Behavior

Females from treatment or control groups were removed from their holding cages and transferred individually to 30 mL cups, each with a ventilated cap. Three healthy males of the same age were cold-immobilized and introduced into each cup containing 1 female. Sexual behaviors, and especially copulation, were observed for 2.5 h, as previously reported by Lietze et al. [9]. If a copulating pair included a hormone-treated female, she was saved for later dissection to confirm viral symptomology. Successful mating acceptance was defined as when females extended their ovipositor and contacted the claspers and aedeagus of the male [13]. Copulation acceptance from the female was indicated when a male and female fly embraced in mating for at least 30 min and did not unclasp from each other. Mating acceptance data were analyzed by G-tests of independence (chi square estimate, [18]) comparing (1) uninfected untreated controls with others; and (2) infected untreated females with others.

## 3. Results

**Figure 1:**
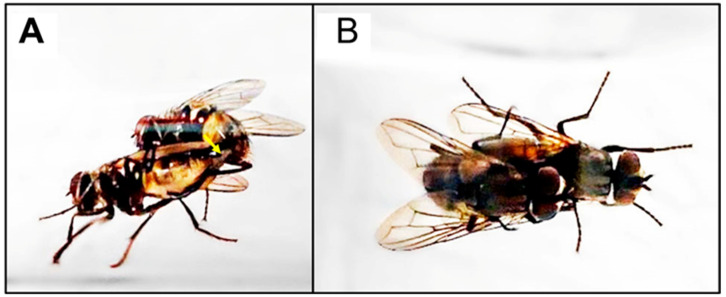
A *Md*SGHV-infected female treated with octopamine, (S)-methoprene, and 20-hydroxyecdysone mated on day 5 with an uninfected, untreated male (**A**,**B**). The copulatory position of the pair is correct, with the male on top (**A**,**B**). The yellow arrow (**A**) points to the female ovipositor that is being drawn into the male’s genital area by his claspers (**A**) while, in (**B**), the normal mating positions is shown, with the male on top.

Successful copulation was observed between infected females treated with hormone therapy and untreated, healthy males (Figure 1). 

Healthy, untreated control females showed a 65% copulation rate for a duration longer than 30 min (Table 1). The mating success of PBS-injected females (80.4%) did not differ significantly from the untreated controls. No copulation was observed in virus-injected flies that were given no hormone therapy. The copulation rates of infected flies that were given octopamine (23%) or methoprene (27.8%) alone were significantly higher than for untreated infected flies, although somewhat lower than for uninfected females. Infected flies that were given both OA and methoprene had copulation success rates (88.9%) that did not differ from uninfected flies. The uninfected flies that fed only on sugar (no protein) did not mate at all, but treatment of these sugar-fed flies with methoprene resulted in mating success (50%) that did not differ significantly from flies that were provided with both sugar and protein (65%).

## 4. Discussion

Our results on the effect of viral infection on female mating receptivity are in broad agreement with those of Leitze et al. [9], who also found that early infection with *Md*SGHV causes females to be refractory to mating attempts by males. The two studies differ somewhat in regard to defining successful copulation. We used the same behavioral observations for successful copulation as described by Tobin and Stoffolano [4] for house flies, which includes the female voluntarily everting her ovipositor so that the male can grasp it and pull it into his genital opening. This contrasts somewhat with Leitze et al. [13], who stated that the female “extended her ovipositor into the genital opening” and that this constituted a successful copulation. In fact, the male grabs and pulls the ovipositor into his genital opening [13].

Manning [19] appears to be the first to have demonstrated the importance of JH in the mating receptivity of females in the Diptera, in this case *D. melanogaster*. Adams and Hintz [20] subsequently discussed how JH stimulates mating in female house flies, while Barth and Lester [21] and Ringo [11,22] later discussed the various factors influencing receptivity in insects and provided references demonstrating that JH is essential for receptivity in many insects (i.e., including flies), as well as that the JH analogue, (S)-methoprene, can induce or restore mating receptivity when given as a hormone replacement therapy. Another important physiological event that can influence mating with respect to JH in flies is when they enter adult diapause [23]. Stoffolano [24] examined the spermathecae of female *Phormia regina* and found that, based on the absence of sperm, females in diapause failed to mate, while non-diapausing females successfully copulated. During their adult diapause, *Phormia regina* and *Protophormia terraenovae* adults refuse to mate [24,25] and, presumably, this is related to the diapause syndrome, which is due to an insufficient amount of JH. Tanigawa et al. [25] were able to rescue CA ablation in *Protophormia* that prevented mating by using a topical application of methoprene. In another study, Teal et al. [12] demonstrated that JH was essential for mating in the Caribbean fruit fly *Anastrepha suspensa* (Loew). 

We found that the JH analog methoprene and octopamine were both effective at rescuing mating receptivity in infected females. In contrast, Kariithi et al. [10] attempted to rescue mating behavior in virus-infected house flies by injecting them with ecdysone, commercial JH-III, or methyl farnesoate, and were unsuccessful in their attempt to produce hormonal therapy. Differences in methodology between the two studies include our use of lower viral doses, the topical application of JH (methoprene), and the use of more than one application of methoprene. 

Adams and Hintz [20] demonstrated that JH was essential for female house flies to accept mating attempts by males, and Yin et al. [14,26] showed that removal of the CA in *P. regina* females significantly reduced receptivity, which could be reversed if they topically applied methoprene. In their paper, Kariithi et al. [10] noted that “*Md*SGHV replication in CA/CC potentially explains the significant reduction of hemolymph sesquiterpenoids levels, the refusal to mate, and the complete shutdown of egg development in viremic females”. The involvement of the CA/CC complex suggests that low or no JH is involved in the lack of mating receptivity in virus-infected female house flies. Evans et al. [15] showed that two applications of 5 µg of methoprene or one 75 µg dose of OA can significantly increase the percentage of insemination in *P. regina* that were fed only sugar, which normally do not mate. OA is a neurohormone that regulates the reproductive function of *Drosophila melanogaster* by controlling the metabolism of JH directly and 20E indirectly [27,28,29,30]. Our results indicate that either JH or OA therapy alone was sufficient to partially restore mating acceptance (23–28%) in virus-infected flies, whereas flies that received both therapies plus 20-hydroxyecdysone (20E) copulated at the same rates (88.9%) as uninfected controls. Further research would be needed to determine whether 20E contributed to the effectiveness of the combination of JH and OA.

The topical application of various juvenoids has been shown to rescue mating receptivity in flies, per the following examples: methoprene for *Protophormia terraenovae* [25], methoprene or fenoxycarb for *Anastrepha suspensa* [12], trans, trans-10, ll-epoxy farnesenic acid, methyl ester for house fly [20], methoprene for *Drosophila melanogaster* [28], OA for *Phormia regina* [15], and now methoprene and octopamine for virus-infected house flies. 

We were able to rescue mating receptivity in virus-infected females. For this study, we applied octopamine by injection, while Barron et al. [31] showed, using honeybees, that various methods of application were suitable. It is possible, as shown by Amsalem et al. [32], that some events in the behavior and physiology of an organism can be rescued by hormone replacement therapy, while other events are unable to be rescued. Hormonal rescue therapy is difficult and can require the application of hormones within a critical window of effectiveness, multiple treatments, an appropriate method of delivery of the treatments, and the tolerance of the study animal to injection. The ability of a therapeutic to rescue a particular pathogen-induced effect may also depend on the dosage of the pathogen or treatment producing the effect.

*Md*SGHV is an attractive biological control agent for managing house flies because of its inhibition of mating behavior and ovarian development. One of the paradoxes of the virus, however, is that flies are only maximally vulnerable to per os infection during a narrow window after adult eclosion, during a time when flies are generally too young to commence feeding [33]. This is thought to be due to development of the peritrophic matrix in the hours after emergence, which prevents the virus from crossing the fly midgut into the hemocoel [34]. House flies are also susceptible to infection by immersion in or sprays with suspensions containing homogenates of virus-infected flies [35]. Although it has limited utility from a fly management standpoint, this viral/house fly system provides a good model to explore the behavioral aspects of how the virus is obtained and spread, the immunity/reproductive tradeoffs, and how it affects mating/copulatory behavior.

## 5. Conclusions

Injecting octopamine and topically applying methoprene twice, following the injection of the virus into healthy females, resulted in the restoration of mating receptivity of infected females. Treatment with octopamine alone showed a lower percentage of mating behavior than treatment with methoprene alone. We demonstrate that methoprene has the greatest effect on rescuing mating behavior in house flies when the treatments are combined. Regardless of hormone treatment, viral injection still resulted in the pathology of the salivary glands and a reduction in ovarian development. The use of the JH-mimic methoprene supports the suggestion that the virus somehow affects sesquiterpenoid production in the corpus allatum or allatotropin from the brain, thus reinforcing JH’s long-understood role in mating receptivity in house fly females. Information is now needed as to whether virus-infected males can be hormonally rescued to mate, and it remains to be determined whether either sex is able to detect virus-infected mates, which might determine mate choice. 

## Figures and Tables

**Table 1:** The effect of various treatments on female house fly mating behavior/copulation. Adult, anautogenous females subjected to various treatments ^a^ and who then mated with uninfected, active mating males. Inf. = females infected with salivary gland hypertrophy and no ovarian development.

Treatment	Dose	# Mated/N ^b^	%Mated ^c^	Chi-Square ^d^	
				Uninfected	Infected
				vs	vs
No injection/no treatment	N/A	17/30	65.0	-	38.154 **
PBS-injected control	2.5 µL	45/70	80.4	0.513	61.564 **
Infected with *Md*SGHV	2.5 µL	0/45	0	33.010 **	-
Inf. + Octopamine (OA)	75 µg	3/20	23.0	9.339 **	6.605 **
Inf. + Methoprene (Meth)	2 × 5 µg	10/60	27.8	14.834 **	11.043 **
Inf. + *Md*SGHV + acetone	2.5 µL + 1 µL	0/10	0	12.652 **	0.102 ns
Inf. + OA, Meth. + 20E	5, 2.5 + 2.5 µg	8/20	88.9	1.340 ns	20.592 **
Sugar-fed only	N/A	0/10	0	12.652 **	0.102 ns
Sugar-fed only + Meth	1 µL	5/10	50.0	0.134	18.722 **

^a^ All treatments were injected except methoprene, which was applied topically. ^b^ Number of females that mated over the total number of females of all trials. #, Number of females that accepted copulation attempts by male flies. ^c^ Percentage of females that copulated with healthy males for a duration longer than 30 min. ^d^ Mating success of either uninfected controls or infected untreated flies compared to others; **, *p* < 0.01, ns, *p* > 0.05.

## Data Availability

Data available upon request from J.G.S.J. or C.J.G.
